# Identification of the Molecular Subtype and Prognostic Characteristics of Breast Cancer Based on Tumor-Infiltrating Regulatory T Cells

**DOI:** 10.1155/tbj/6913291

**Published:** 2025-03-05

**Authors:** Jianying Ma, Gang Hu, Lianghong Kuang, Zhongzhong Zhu

**Affiliations:** ^1^Department of Breast Surgery, Thyroid Surgery, Huangshi Central Hospital, Affiliated Hospital of Hubei Polytechnic University, Huangshi, Hubei, China; ^2^Department of Breast Surgery, Thyroid Surgery, Wuhan Third Hospital, Tongren Hospital of Wuhan University, Wuhan, Hubei, China; ^3^Department of Neurology, Huangshi Central Hospital, Affiliated Hospital of Hubei Polytechnic University, Huangshi, Hubei, China; ^4^Department of Gastroentero Rectal Surgery, Huangshi Central Hospital, Affiliated Hospital of Hubei Polytechnic University, Huangshi, Hubei, China

**Keywords:** breast cancer, immunotherapy, prognostic model, regulatory T cells, tumor microenvironment

## Abstract

**Background:** T regulatory cells (Tregs) are essential for preserving immune tolerance. They are present in large numbers in many tumors, hindering potentially beneficial antitumor responses. However, their predictive significance for breast cancer (BC) remains ambiguous. This study aimed to explore genes associated with Tregs and develop a prognostic signature associated with Tregs.

**Methods:** The gene expression and clinical data on BC were obtained from The Cancer Genome Atlas (TCGA) and Gene Expression Omnibus (GEO) databases. The integration of CIBERSORT and weighted correlation network analysis (WGCNA) algorithms was utilized to identify modules associated with Tregs. The consensus cluster algorithm was utilized to create molecular subtypes determined by genes associated with Tregs. Then, a prognostic signature associated with Tregs was constructed and its relationship to tumor immunity and the prognosis was evaluated.

**Results:** The blue module genes exhibited the most significant correlation with Tregs, and 1080 genes related to Tregs were acquired. A total of 93 genes from the TCGA dataset were found to have a significant impact on patient prognosis. Samples from BC were categorized into two clusters by consensus cluster analysis. The overall survival, immune checkpoint genes, molecular subtype, and biological behaviors varied significantly between these two subtypes. A 10-gene signature developed from differentially expressed genes between two subtypes demonstrated consistent prognostic accuracy in both TCGA and GEO datasets. It functioned as a standalone prognostic marker for individuals with BC. In addition, patients with low risk are more inclined to exhibit increased immune cell infiltration, TME score, and tumor mutation burden (TMB). Meanwhile, Individuals classified within the low-risk group showed better responses to immunotherapies compared to their counterparts in the high-risk group.

**Conclusions:** The prognostic model derived from Tregs-related genes could aid in assessing the prognosis, guiding personalized treatment, and potentially enhancing the clinical outcomes for patients with BC.

## 1. Introduction

Breast cancer (BC) is a prevalent form of cancer in females and a major contributor to female deaths globally [1]. Recent studies show that there are about 2.3 million new cases diagnosed each year, resulting in a mortality rate of approximately 450,000 [2, 3]. BC can be categorized into five major subtypes (Luminal A, Luminal B, HER2-enriched, normal-like, and basal-like) by examining the immunohistochemical expression of specific receptors (estrogen [ER], progesterone [PR], and HER2 receptors) [4]. ER + BC is typically treated with endocrine therapies either by themselves or in combination with targeted therapies such as CDK 4/6 inhibitors. Individuals diagnosed with HER2+ tumors receive treatment involving antibodies or antibody–drug conjugates and tyrosine kinase inhibitors that target HER2. BRCA–mutant BC has additional treatment options with PARP inhibitors. Tailored therapies for triple-negative BC (TNBC) are limited, as it is the most aggressive subtype and is typically treated with chemotherapy alone or in conjunction with immunotherapy. The few choices available for treating TNBC, along with the emergence of resistance to treatments for ER+, PR+, and HER2+ BC, underscore the significant therapeutic challenges that still exist [1]. This emphasizes the necessity for a deeper insight into the molecular mechanisms that drive the progression of BC.

The tumor microenvironment (TME) consists of immune cells, and other nonimmune components (fibroblasts and extracellular matrix), all of which surround cancer cells. The active interplay between tumor cells and TME can hinder the immune system's ability to monitor tumor cells, speed up the growth of tumor cells, promote immune evasion and resistance to treatment, and have a crucial impact on the development and advancement of tumors [5]. Increasing evidence suggests that immune cells infiltrating tumors (TIICs) are involved in the progression and advancement of cancer [6]. TIICs within the TME can impact both drug responsiveness and cancer prognosis [7]. Regulatory T cells (Tregs), which are a specialized subgroup of CD4+ T lymphocytes, are essential for maintaining immune balance by controlling responses to infections, allergies, and organ transplants, while also inhibiting protective immune reactions [8, 9]. Tregs control the activation and development of typical CD4+ T cells, along with numerous other cell types in both the innate and adaptive immune systems, using various effector mechanisms [10]. By releasing proangiogenic substances, Tregs support the formation of new blood vessels, ensuring a continuous supply of nutrients for tumor growth [11]. In addition, interactions between Tregs and other TIICs, such as myeloid-derived suppressor cells, establish a collaborative immunosuppressive network that promotes tumor immune escape [12]. Tregs are frequently present in higher numbers in tumors compared to blood or lymphoid organs. Increasing evidence has suggested that a high concentration of Tregs is correlated with the worse clinical outcomes of solid tumors [13–15]. Recently, the application of immunotherapy involving ICIs, specifically CTLA-4 and PD-1, has shown encouraging results in treating cancer, possibly due to their effect on Tregs and enhanced destruction of Teff cells [9, 16]. The relationship between Tregs infiltration and PD-1 expression is both close and intricate. In cancer patients, the presence of Tregs that express PD-1 in the TME can affect immune suppression and correlate with disease advancement [17]. Blocking PD-1 leads to increased immunosuppression mediated by PD-1+ Tregs [18]. Furthermore, lenvatinib has been shown to enhance the efficacy of anti-PD-1 therapy through the reduction of tumor PD-L1 levels and inhibition of Tregs differentiation [19]. Hence, delving further into these aspects can enhance our comprehension of the intricate processes within the TME during BC progression, aiding clinicians in devising effective approaches for utilizing ICIs in treating cancer.

Bioinformation technology advancements have led to the utilization of numerous techniques for identifying biomarkers. The weighted correlation network analysis (WGCNA) algorithm is capable of detecting modules and genes that are strongly correlated in cancer by analyzing the gene expression network. Datasets were collected in this study to analyze immune cell expression patterns and identify marker genes linked to Tregs through WGCNA. Various molecular categories were identified, and a predictive risk model was created and validated based on gene expression data from public databases. Furthermore, the association of the prognostic signature with the TME, mutation, immunotherapy, and chemotherapy was examined. Our systematic examination of Treg cell populations in BC patients establishes a foundation for clinical outcomes and potential immunotherapy applications.

## 2. Materials and Methods

### 2.1. Data Extraction and Processing

The transcriptome data pertaining to 1073 BC tissue samples, and their associated clinicopathological traits, were obtained from The Cancer Genome Atlas (TCGA) database, accessible at https://portal.gdc.cancer.gov/repository. Moreover, the gene expression information of BC tissues along with their related clinical characteristics were obtained from the Gene Expression Omnibus (GEO) repository (https://www.ncbi.nlm.nih.gov/geo/, GSE20685, GSE21653, and GSE22219). The analysis was performed using the TCGA dataset along with three GEO datasets, which served as both the training and validation cohorts.

### 2.2. Discovery of Marker Genes for Tregs

The proportions of 22 different types of TIICs in BC samples were determined using the CIBERSORT algorithm. Next, we conducted a WGCNA to identify genes associated with Tregs. Initially, a coexpression network was constructed using the TCGA dataset expression data and the “WGCNA” R package. The pickSoftThreshold function was utilized to calculate *β* values to determine the most suitable soft threshold for creating a scale-free network. Following this, the gene correlation matrix was converted into a weighted matrix by the best soft threshold, which was then used to generate an adjacency matrix. A topological overlap matrix (TOM) was computed from the adjacency matrix to show the shared neighbors' overlap. Furthermore, the gene dendrogram and module color were established based on the TOM–based dissimilarity measure with a minimum gene number of 200. In addition, we divided the main module using Dynamic Tree Cut and merged modules with similar characteristics. Next, we evaluated the connections between modules and the presence of TIICs through Pearson's correlation test. Genes from these particular modules that exhibited a significant relationship with Tregs were chosen for further analysis.

### 2.3. Cluster Analysis for Tregs-Related Genes

The module associated with the infiltration of Tregs was selected and the genes within it underwent univariate Cox regression analysis. By employing the expression matrix of prognostic genes linked to Tregs in BC patients, we conducted a cluster analysis utilizing the “ConsensusClusterPlus” R package. The cumulative distribution function was utilized to determine the most appropriate number of clusters. Survival rates among the various clusters were analyzed through the Kaplan–Meier method, and biological distinctions between the clusters were assessed using gene set variation analysis (GSVA). Furthermore, we analyzed the differences in immune checkpoint expression across the distinct clusters.

### 2.4. Construction and Validation of a Prognostic Model Based on Tregs-Related Genes

Differentially expressed genes (DEGs) across different subtypes were identified using the “limma” R package. DEGs with a false discovery rate (FDR) threshold of < 0.05 and |log_2_ fold change (FC)| of > 0.5 were considered statistically significant. By integrating the prognostic data of patients with univariate analysis of the identified DEGs, we successfully determined the OS-associated genes among the DEGs. Next, the prognostic model is built using regression analysis with LASSO and multivariate Cox regression. Risk scores for individual BC patients are determined by evaluating the expression levels of DEGs (Expi) and Cox coefficients (coefi) as risk score = ∑_*i*=1_^*n*^Exp*i* × coef*i*. According to the optimal cutoff value, patients from the TCGA and GEO cohorts were stratified into high- and low-risk categories separately. The prognosis of each group is evaluated using the Kaplan–Meier curve to determine the overall survival. The risk model's predictive accuracy is assessed through time-dependent ROC analysis with the “survivalROC” package. The correlation between risk score and clinical traits was analyzed. Univariate and multivariate Cox analyses were employed to estimate whether the risk score is an independent indicator of clinicopathological features. Nomogram plots integrating risk score and clinicopathological traits were performed with the “rms” package. Calibration plots and time-dependent ROC curves were used to assess the effectiveness of the nomogram.

### 2.5. Immune Infiltration Analysis

The CIBERSORT technique was utilized to assess the levels of 22 different TIICs in every sample within the TCGA database, while also examining how these immune cell levels varied based on different risk score groups. Using single-sample gene set enrichment analysis (ssGSEA), we analyzed immune cell infiltration and immune function in BC samples. Moreover, the TME score (immune score and stromal score) of every sample was calculated with the “estimate” R package, and the disparities in TME among various risk groups were analyzed.

### 2.6. Comparison of Immunotherapy and Chemotherapy for Different Risk Groups

The variations in the effects of immunotherapy and chemotherapy between the different risk groups were analyzed. The TIDE score was computed for every sample to evaluate the potential clinical effects of immunotherapy in two risk groups on the TIDE website (https://tide.dfci.harvard.edu/). An elevated TIDE score suggests an increased probability of immune evasion, signifying that patients may have reduced chances of benefiting from immunotherapy. The immunophenoscore (IPS) of BC patients was obtained from The Cancer Immunome Atlas (TCIA) database (https://tcia.at/home/). Individuals who have elevated IPS levels have an increased probability of reacting positively to ICIs. The IPS variances among various risk groups were analyzed. In addition, an examination was conducted on the variations in the manifestation of genes associated with antigen presentation and immune checkpoints among different risk categories. The TCGA database offers the original data on tumor mutation burden (TMB) for the samples. Initially, the research obtained the initial TMB data for each BC sample and then computed the TMB value for each sample.

We employ the GDSC and CCLE online databases to gather information on how tumor cells react to drugs. The “oncoPredict” package is utilized to assess the responsiveness of individual samples in the TCGA set to drugs, using information from the GDSC V2.0 repository.

### 2.7. Functional Enrichment Analysis

The “limma” package was used to determine the DEGs between the low and high groups (|log_2_FC| > 1.5 and FDR < 0.05). DEGs were analyzed employing the R package “clusterProfiler” for pathways and biological processes'6 enrichment analysis including GO analysis and the KEGG.

### 2.8. Statistical Analysis

R (Version 4.3.0) was utilized for all statistical analyses in this investigation. The Wilcoxon test was employed to assess the disparities between the two groups. Within the framework of the WGCNA analysis, Pearson's correlation test was employed to evaluate the correlation coefficients between the feature genes of each module and TIICs. Both univariate and multivariate Cox regression analyses were conducted using the R package “survival.” Kaplan–Meier survival curves were plotted, and survival differences were compared via the log-rank test. For detecting differences, a *p* value less than 0.05 was considered statistically significant.

## 3. Results

### 3.1. Discovery of Genes Linked to Tregs Using WGCNA

**Figure 1:**
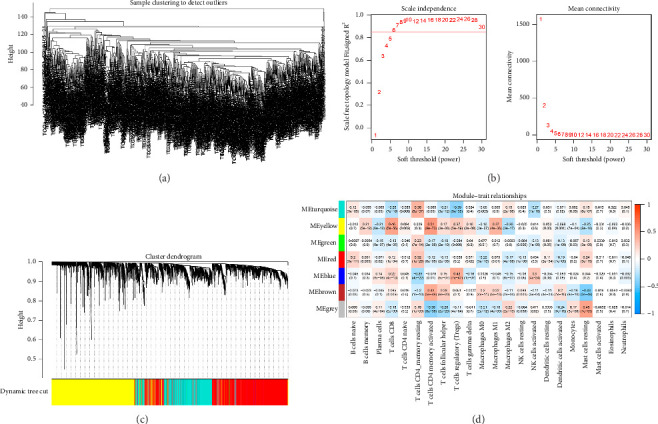
Discovery of genes linked to Tregs through WGCNA. (a) Sample clustering analysis to detect outliers. (b) Analysis of the network topology for various soft-thresholding powers. (c) The cluster dendrogram with the gene modules and module merging. (d) The correlations between gene modules and immune cells.

First, we calculated the proportions of 22 TIICs in 1073 BC samples based on the CIBERSORT algorithm. Then, we conducted a hierarchical clustering analysis on the expression patterns of these samples (Figure 1(a)). The optimal soft threshold power was determined using the pickSoftThreshold function, resulting in an *R*^2^ value of around 0.86 at a power of 6 (Figure 1(b)). With the assistance of the Dynamic Tree Cut package, we identified 7 modules (Figure 1(c)). The blue module showed the strongest positive correlation with Tregs, while its connection with other immune cells was minimal (Figure 1(d)). Thus, 1080 genes in the blue module were considered Tregs-related genes.

### 3.2. Cluster Analysis for Tregs-Related Genes

**Figure 2:**
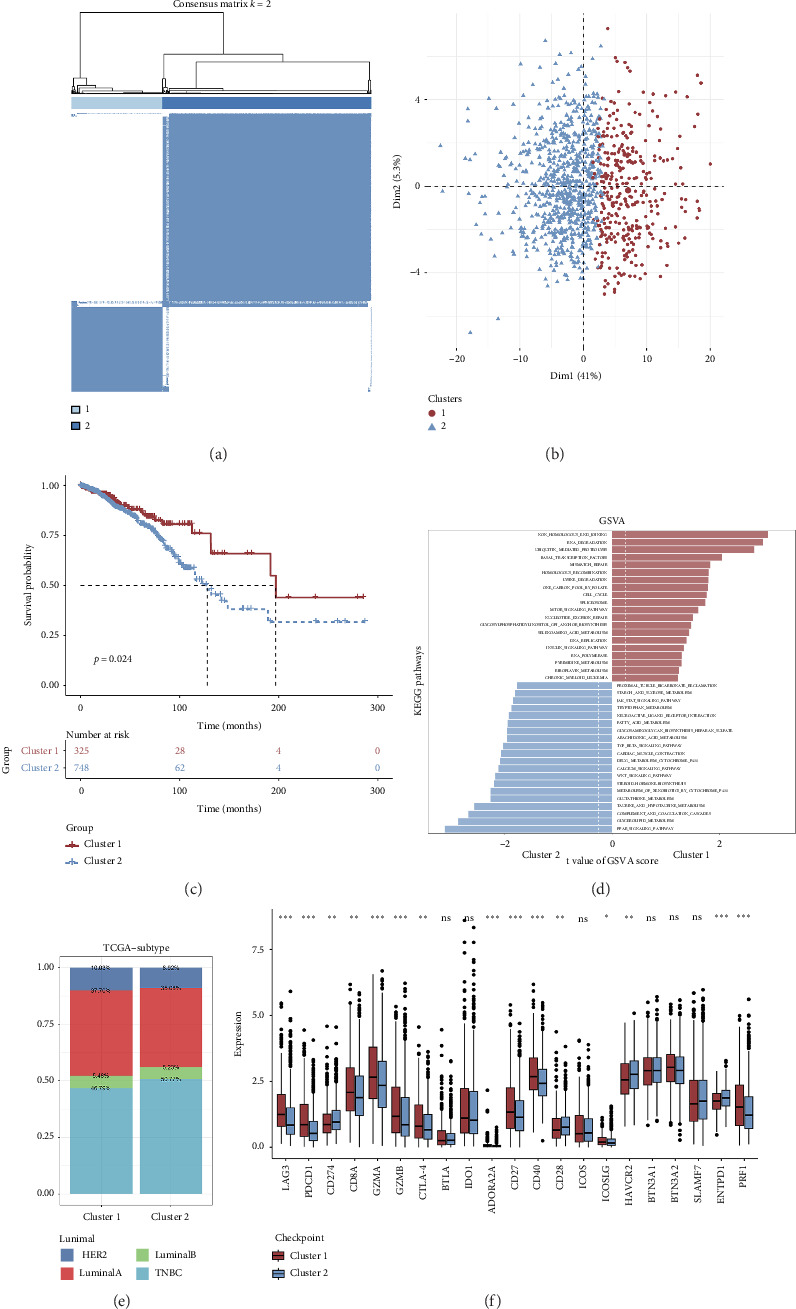
Cluster analysis for Tregs-related genes. (a) Consensus clustering analysis revealed the presence of two molecular clusters associated with Tregs. (b) The PCA analysis of distinct Tregs-related molecular clusters. (c) The comparison of survival rates among the two clusters. (d) The variances in the biological behaviors of two clusters by GSVA analysis. (e) Percentage of patients in two clusters with various molecular subtypes of BC. (f) The variations in the expression of genes related to immune checkpoints among the two clusters.

Initially, we conducted a univariate survival analysis on genes associated with Tregs using data from TCGA. The results revealed that 93 genes were linked to the prognosis of BC in TCGA (Supporting Table S1). Based on the expression of prognostic genes related to Tregs, we performed a cluster analysis on BC samples from the TCGA cohort using the “ConsensusClusterPlus” R package, which classified these patients into two clusters: 1 and 2 (Figure 2(a)). Principal component analysis (PCA) confirmed the excellent intergroup distribution among the two clusters (Figure 2(b)). The analysis of clinical survival data revealed that Cluster 1 demonstrated a markedly improved OS in comparison to Cluster 2 (Figure 2(c)). The GSVA analysis was conducted on the variances in biological processes between the two clusters. The findings demonstrate that the two subtypes exhibit considerable disparities in their biological characteristics (Figure 2(d)). Cluster 2 has higher scores in the cell cycle, JAK/STAT, TGF-beta, and Wnt signaling pathways compared to Cluster 1 (Figure 2(d)). Based on the molecular subtypes of BC, we found a higher proportion of TNBC subtypes in Cluster 2 (50.77% vs. 46.79%) than in Cluster 1 (Figure 2(e)). In addition, we examined the immune checkpoint expression in two clusters. Upon investigation, it was found that 16 out of the 22 immune checkpoints showed significant variations among the two clusters (Figure 2(f)).

### 3.3. Development and Validation of the Tregs-Related Prognostic Model

**Figure 3:**
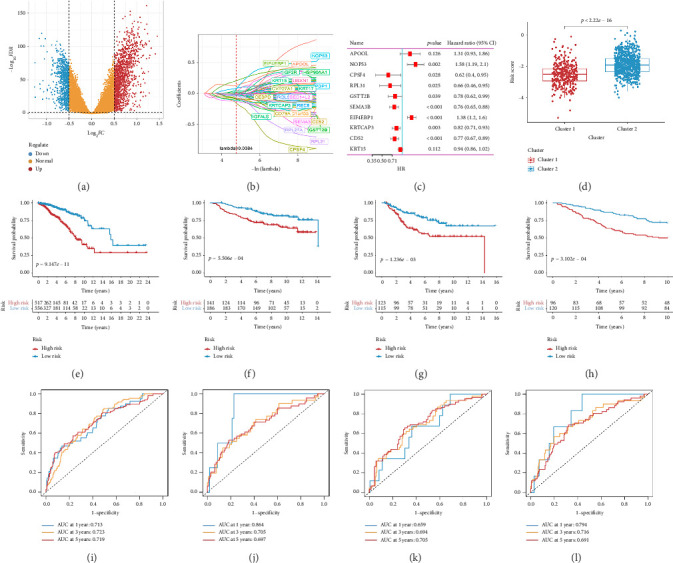
Development and validation of the Tregs-related prognostic model. (a) Volcano plot of the differentially expressed genes (DEGs) between two clusters. (b) Coefficients of the LASSO analysis. (c) Multivariate Cox regression analysis of 10 prognostic DEGs. (d) Difference in the risk scores among the two clusters. (e–h) Differences in overall survival of BC patients in training cohort (e), GSE20685 cohort (f), GSE21653 cohort (g), and GSE22219 cohort (h). (i–l) Time-dependent ROC curves analysis in training cohort (i), GSE20685 cohort (j), GSE21653 cohort (k), and GSE22219 cohort (l).

Utilizing a FDR threshold of less than 0.05 and an absolute log2FC exceeding 0.5, we screened 2088 DEGs between two clusters (Figure 3(a)). Through the application of univariate Cox regression analysis, 82 prognosis-related DEGs were screened in the TCGA training set. Consequently, the LASSO–penalized Cox analysis also pinpointed 10 DEGs for further multivariate analysis (Figure 3(b)). The multivariate Cox model was constructed incrementally by employing the likelihood-ratio forward approach to achieve the greatest level of significance (Figure 3(c)). A set of 10 DEGs were selected for the development of a prognostic signature. Risk score = 0.272∗APOOL+0.457∗NOP53 − 0.484∗CPSF4 − 0.413∗RPL31 − 0.248∗GSTT2B − 0.279∗SEMA3B+0.324∗EIF4EBP1 − 0.203∗KRTCAP3 − 0.259∗CD52 − 0.067∗KRT15. Expression levels from the training and three validation datasets were utilized to compute the risk score for each sample. The comparative assessment of scores across various clusters indicated that Cluster 2 exhibited significantly elevated scores in comparison to Cluster 1 (Figure 3(d)). The samples were stratified into two risk categories based on the established optimal cutoff value. A survival analysis of the distinct risk score groups demonstrated that the individuals with low-risk scores experienced considerably superior survival rates compared to those with high-risk scores within the training cohort (*p* < 0.001), GSE20685 cohort (*p* < 0.001), GSE21653 cohort (*p*=0.001), and GSE22219 cohort (*p* < 0.001) separately (Figures 3(e), 3(f), 3(g), and 3(h)). Furthermore, the model showed high efficiency in predicting prognosis at 5 years, with the AUC of 0.719, 0.697, 0.705, and 0.691, respectively (Figures 3(i), 3(j), 3(k), and 3(l)).

### 3.4. Formation of a Nomogram Model

**Figure 4:**
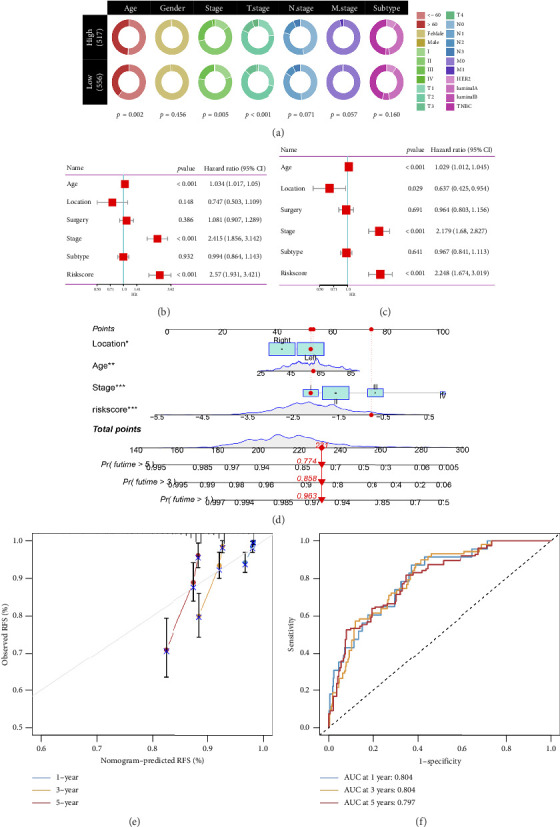
Development and validation of a novel nomogram in the TCGA cohort. (a) The correlation between risk score and clinic–pathologic feature. (b) Univariate and multivariate (c) cox analyses. (d) A prognostic nomogram predicting 1-, 3-, and 5-year overall survival of BC. (e) The calibration curve of the nomogram predicts the 1-, 3-, and 5-year survival rate. (f) ROC analysis showing the AUCs of nomogram in predicting the 1-, 3-, and 5-year overall survival.

The relationship between risk score and clinic–pathologic features was analyzed. The high-risk score group was correlated with those over 60 years old and advanced TNM stage (Figure 4(a)). Furthermore, both univariate and multivariate examinations of age, location, surgery, TNM stage, subtype, and risk score indicated that the risk score independently predicted the prognosis of BC (Figures 4(b) and 4(c)). A prognostic nomogram was created to predict OS in BC patients using the TCGA dataset to guarantee the strength and usefulness of the prognostic model (Figure 4(d)). The nomogram incorporated variables such as age, location, stage, and risk scores. Internal validation of the nomogram was performed through the generation of a calibration plot (Figure 4(e)). Moreover, the ROC curve analysis demonstrated that the scores derived from the nomogram exhibited strong predictive capabilities regarding the patient's OS, as evidenced by elevated AUC values at the 1-, 3-, and 5-year intervals (Figure 4(f)).

### 3.5. Immune Infiltration Differences Between Groups

**Figure 5:**
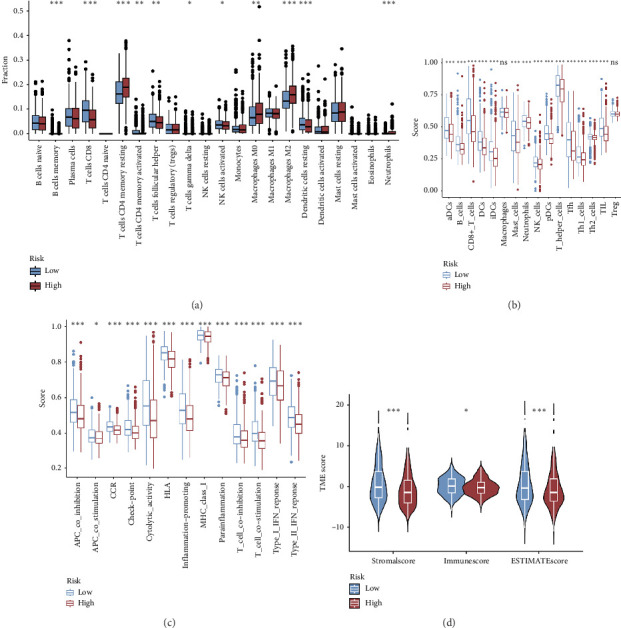
Immune infiltration differences between high- and low-risk groups. (a) Correlation between the risk score and immune infiltrating cells by the CIBERSORT algorithm. (b) Differences between immune infiltrating cells and immune function (c) between the two risk groups by the ssGSEA algorithm. (d) Variations in the TME score among the two risk groups.

The CIBERSORT method was applied to the TCGA dataset to assess the quantities of 22 distinct TIICs in each sample. As shown in Figure 5(a), the infiltration levels of B cells memory, CD8 T cells, resting and activated memory CD4 T cells, and activated NK cells were higher in the low-risk group than those in the high-risk group, whereas the high-risk group showed significantly higher infiltration of M0 and M2 macrophages compared to the low-risk group. In addition, the ssGSEA approach was utilized to assess the abundance of immune cell populations and the functionalities associated with the immune response in the two distinct risk categories. In the low-risk group, most immune cells are significantly more abundant than in the high-risk group (Figure 5(b)). Simultaneously, we noted that the immune responses in the low-risk group were notably more heightened when juxtaposed with the high-risk group (Figure 5(c)). The outcomes of the TME analysis indicated that the stromal, immune, and overall ESTIMATE scores were elevated in the low-risk group when contrasted with the high-risk group (Figure 5(d)).

### 3.6. Differences in Immunotherapy and Chemotherapy Responses Between Groups

**Figure 6:**
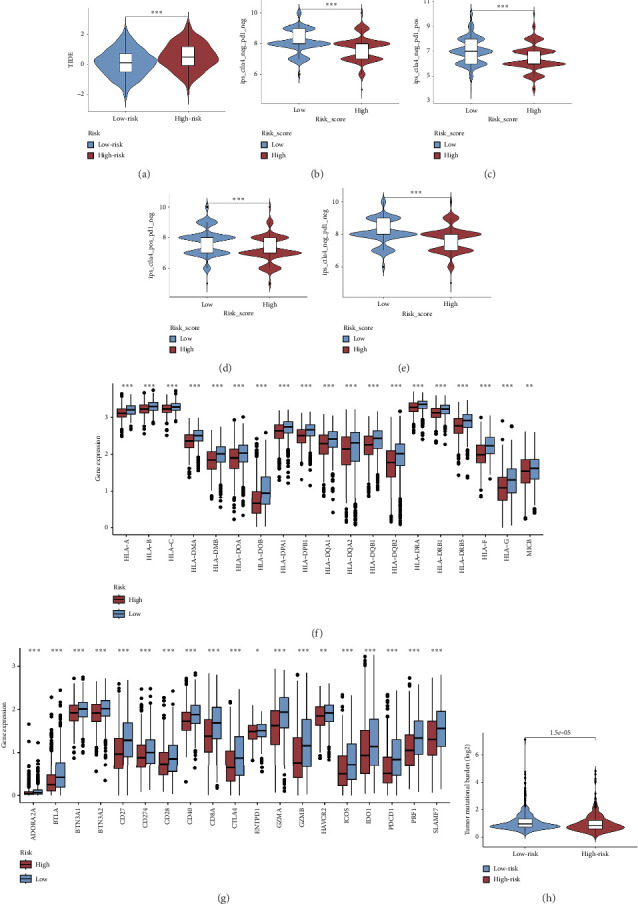
Differences in immunotherapy responses between the groups. (a) Comparison of the TIDE score in the two risk groups. (b–e) Differences in IPS among the two risk groups. (f) Differences in the expression of antigen presentation and (g) immune checkpoint genes. (h) Differences in TMB among the two risk groups.

**Figure 7:**
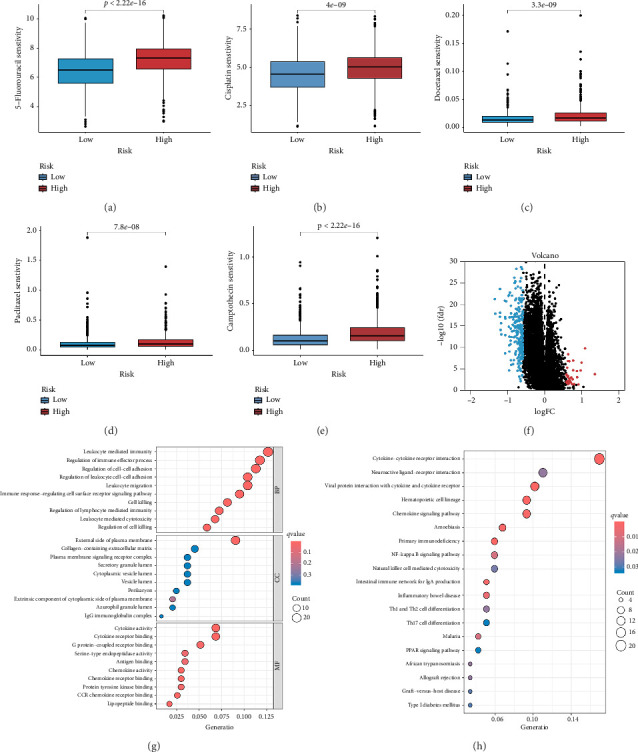
Chemotherapeutic sensitivity and functional enrichment analysis. (a–e) The box plots of the estimated IC50 for four drugs between the high- and low-risk groups. (f) Volcano plot of the DEGs. (g) Dot plot of GO terms, including BP, CC, and MF analysis. (h) Dot plot of the KEGG enrichment analyses.

We examined the variations in responses to immunotherapy and chemotherapy between the two risk categories. To assess the possible clinical implications of immunotherapy, we utilized the TIDE software. A greater TIDE forecast rating indicates an increased likelihood of immune evasion, leading to decreased possibilities of benefiting from immunotherapy for patients. In the TCGA datasets, the high-risk group exhibited a markedly elevated TIDE score in comparison to the low-risk group (Figure 6(a)). The IPS of four groups in the high-risk group showed a notable decrease (Figures 6(b), 6(c), 6(d), and 6(e)), suggesting reduced immune response to ICIs in the high-risk group. Furthermore, the low-risk score group exhibited significantly elevated expression levels of antigen presentation and immune checkpoint–related genes compared to the high-risk score group (Figures 6(f) and 6(g)). Furthermore, the TCGA mutation dataset was processed using Mutect2 software to calculate the TMB for patients and analyze the TMB distribution among the two risk groups. We found that TMB was significantly elevated in the low-risk group as compared to the high-risk group (Figure 6(h)). The sensitivity of each sample to chemotherapeutic drugs was estimated utilizing the “oncoPredict” R package. The findings indicated that the IC50 of 5-fluorouracil, cisplatin, docetaxel, paclitaxel, and camptothecin was notably reduced in the low-risk group compared to the high-risk group, implying that patients with lower risk scores may derive greater benefits from these medications (Figures 7(a), 7(b), 7(c), 7(d), and 7(e)).

### 3.7. Functional Enrichment Analysis

A total of 263 DEGs were identified between low and high groups with the threshold of |log_2_FC| > 1.5 and FDR < 0.05 (Figure 7(f)). GO analysis demonstrated that signature is markedly associated with various essential biological processes related to cancer, such as leukocyte-mediated immunity, regulation of immune effector process, and immune response–regulating cell surface receptor signaling pathway (Figure 7(g)). In addition, the risk score is associated with various crucial KEGG pathways, including natural killer cell–mediated cytotoxicity, NF-kappa B signaling pathway, and Th1 and Th2 cell differentiation (Figure 7(h)).

## 4. Discussion

BC is a highly heterogeneous tumor, and its genetic and molecular characteristics, as well as TME, are critical to cancer progression and sensitivity to therapeutics [20]. In recent years, the TME has attracted considerable interest owing to its pivotal involvement in tumor immunosuppression, metastatic dissemination, and resistance to therapeutic agents [21]. The TME is characterized by its immune surveillance and defense mechanisms against neoplastic cells. In addition, inflammation associated with tumors can lead to irregular accumulation of immune cells into tumor tissue and its vicinity, which disrupts the equilibrium of chemokine and cytokine production. This disruption facilitates the ability of tumor cells to evade immune detection, ultimately fostering tumor progression [22–24]. The TME comprises a variety of elements, including neoplastic cells, and TIICs, along with endothelial and stromal constituents. The complex interactions among these components can progressively alter the TME to a condition that inhibits the immune response of the host [25]. The fragile equilibrium between antitumor and protumor inflammatory factors is crucial in determining tumor development and the effectiveness of antitumor immune responses. A growing body of evidence indicates that the TME significantly influences the onset and advancement of BC, as well as the resistance to drugs and the effectiveness of immunotherapy [26–28]. Thus, it is crucial to have a comprehensive grasp of the precise process of TME in the advancement of BC to effectively strategize and develop tailored treatments for BC.

Within the TME, Tregs are a major therapeutic target. Current research in the field is focused on Tregs that inhibit anticancer immunity by expressing the key transcription factor Foxp3, which hinders the protective monitoring of tumors and impairs effective immune responses against tumors in hosts with tumors [8, 29]. Tregs are primarily utilized as possible targets for suppressing the activation and differentiation of CD4+ helper T cells and CD8+ cytotoxic T cells, as well as for promoting reactivity to self and tumor-specific antigens [30, 31]. Within the TME, Tregs can be generated and transformed by conventional T cells, possessing potent immunosuppressive capabilities that hinder anticancer defenses and facilitate tumor progression. Tregs play a crucial role in tumor immune evasion by inhibiting the activity of immune effector cells through various mechanisms [32, 33]. There is a growing evidence indicating that reducing Treg cells leads to a rise in the amount of CD4+ and/or CD8+ effector T cells (Teffs) within tumors, enhancing the body's ability to fight against tumors and resulting in tumor rejection [34]. Therefore, eliminating tumors in these environments was partially attributed to the elimination of Tregs-induced inhibition of the immune response against tumors. Likewise, the efficacy of various cancer immunotherapies approved by the FDA, such as anti-CTLA-4 and anti-PD-1, could be linked to their impact on Tregs as well as enhancing Teff cell destruction [35, 36]. Tumor growth and advancement are heavily influenced by the inhibition of immune responses against cancer cells by Tregs. Depleting Tregs in the TME may become a novel strategy of BC immunotherapy. Therefore, it is imperative to find Tregs-specific markers for cancer treatment.

A recently published study has focused on the role of Tregs in BC and developed a regulatory T cell-associated model that can predict patient prognosis and response to therapy. Although there are some similarities between this study and ours, however, they differ in their methodologies, findings, and implications. The following shows the highlights and innovations of our study. First, advanced analytical techniques were used to screen Tregs-related genes in this study. The use of CIBERSORT and WGCNA to identify modules associated with Tregs represents a sophisticated approach to analyzing gene expression data. This methodology allows for a nuanced understanding of the relationship between Tregs and other genes, which may not be fully captured in simpler analyses. Second, consensus clustering of molecular subtypes was carried out in this study. The application of consensus clustering to stratify patients into molecular subtypes according to Tregs-associated genes is an innovative approach. This classification can reveal distinct biological behaviors and prognostic outcomes, offering insights into the heterogeneity of BC. Third, several databases were integrated in this study to verify the accuracy of the signature. Our study utilizes data from both TCGA and three GEO datasets, enhancing the robustness of the findings through cross-validation across different datasets. Fourth, the value of the signature for predicting immunotherapy response was explored in this study. The findings suggest that individuals with low-risk scores exhibit better responses to immunotherapies, highlighting the potential for personalized treatment strategies based on Tregs-related gene signatures. This aspect is particularly relevant given the increasing importance of immunotherapy in BC treatment. Taken together, our study provides significant advancements in understanding the role of Tregs in BC through innovative methodologies and comprehensive analyses. Its focus on molecular subtyping, immune microenvironment interactions, and the development of a robust prognostic model contributes valuable insights for personalized treatment approaches.

The objective of this study was to identify genes associated with Tregs and analyze their clinical significance in BC using transcriptome sequencing data. We utilized the CIBERSORT method to calculate the relative abundance of 22 distinct TIICs. Subsequently, a WGCNA was built to select the gene modules most closely associated with Tregs. The blue module was chosen as the most pertinent one, with its genes being identified as Tregs-associated genes. Utilizing these Tregs-related genes, we identified two distinct molecular clusters related to Tregs, which exhibited significant disparities in prognosis, molecular subtype, expression of important immune checkpoints, and biological functions. With poorer OS, Cluster 2 was characterized by an abundance of the tumor-related pathway and a deficiency of immune response pathways. Furthermore, the BC can be categorized into HER2-enriched, Luminal A, Luminal B, and TNBC subtypes. In contrast to Cluster 1, Cluster 2 exhibited a greater percentage of TNBC subtype. These elements constitute a significant contributor to the unfavorable prognosis observed in patients categorized within Cluster 2 when contrasted with those in Cluster 1. Based on the DEGs between the two Tregs-related clusters, we developed a Tregs cluster–related prognostic signature and generated a risk score.

Within the TCGA group, Cluster 2 exhibited a notably elevated risk score in comparison to Cluster 1, leading to a marked reduction in overall survival within the high-risk score category when contrasted with the low-risk score category. The signature is capable of accurately stratifying the survival of BC patients. We tested the accuracy of the signature by performing validation both internally and externally. The effectiveness of the signature was outstanding in both groups. Multivariate analysis demonstrated that the Tregs-associated signature serves as an independent indicator for OS in BC. To better adapt this model for clinical use, a nomogram was developed that incorporates both the risk score and clinical characteristics. Collectively, this offered a new method for forecasting outcomes and presented fresh insights into the enigmatic functions of Tregs within the BC microenvironment on a transcriptome scale.

In recent years, remarkable progress has been achieved in the field of cancer immunotherapy, particularly through the development and application of immunotherapeutic agents that primarily focus on the CTLA-4, PD-1, and PD-L1 immune checkpoint pathways [37]. Patients with inoperable tumors have increased life-extending options with immunotherapy [38]. Tumor therapy relies heavily on a thorough knowledge of the immune landscape of the TME. Given that many BC patients continue to experience a poor outlook even after undergoing immunotherapy due to immune evasion or immune tolerance [39], we investigated the immune environment of BC using the Tregs-associated risk signature. The study revealed that individuals in the high-risk category exhibited reduced infiltration of CD8 + T cells alongside higher levels of M2 macrophage infiltration when compared to those in the low-risk category. Research has indicated that M2 macrophages contribute to the facilitation of immune tolerance during cancer immunotherapy [40]. To further explore the correlation between risk score and immunotherapy response in BC, we applied several scores. A high TMB score is a strong indicator of improved response to immunotherapy [41]. The findings of our study showed a notable increase in TMB levels among individuals in the low-risk category in comparison to those in the high-risk category. A decreased TIDE score indicated a reduced chance of immune evasion and a more favorable reaction to immunotherapy [42]. Individuals classified with low-risk scores exhibited reduced TIDE scores in comparison to their counterparts in the high-risk score group. The IPS was a more accurate indicator of how well patients would respond to treatments with anti-CTLA4 and anti-PD-1 antibodies, with a higher IPS suggesting a more positive outcome from immunotherapy [43]. Patients with a low-risk score exhibited elevated IPS levels, indicating a potentially more favorable response to ICIs. Previous studies have established a connection between the obstruction of antigen presentation and mechanisms of immune evasion. Antigen presentation plays a crucial role in initiating the immune response directed toward tumors. Our results revealed that individuals categorized within the high-risk score group demonstrated a significant reduction in antigen presentation compared to those in the low-risk score group, implying a relationship between an elevated risk score and a weakened response to immunotherapeutic interventions. In addition, our analysis revealed a lower expression of immune checkpoint genes and ligand receptors in the high-risk group versus the low-risk group. Synthesizing these findings, we deduce that patients classified in the low-risk group are more likely to derive benefits from immunotherapy.

## 5. Conclusions

We screened 93 Tregs-related prognostic genes and identified two distinct molecular subtypes of BC with notably significant prognoses, biological behaviors, and immunotherapy effects. In addition, the 10-gene signature we established exhibited stable performance in predicting the prognosis and response to immunotherapy of BC patients and possessed potential clinical utility.

## Data Availability

The data used to support the findings of this study are available from the corresponding author upon request.

## Supporting Information

Table S1: Univariate Cox analysis of genes associated with Tregs in the TCGA cohort. Table S1 shows 93 TREGs-related prognostic genes obtained through univariate analysis.
