# Supplementary material for: Identification of the Molecular Subtype and Prognostic Characteristics of Breast Cancer Based on Tumor-Infiltrating Regulatory T Cells
Source: Breast J. 2025 Mar 5;2025:6913291. doi: 10.1155/tbj/6913291 (PMC11991805; doi:10.1155/tbj/6913291)
Supplement: Supporting Information — Additional supporting information can be found online in the Supporting Information section. [file 6913291.f1.docx]

**Table S1 Univariate Cox analysis of genes associated with Tregs in the TCGA cohort.** Table S1 shows 93 TREGs-related prognostic genes obtained through univariate analysis.

| **Gene** | **P-value** | **HR** | **Low 95%CI** | **High 95%CI** | **coef** | **Type** |
| --- | --- | --- | --- | --- | --- | --- |
| TNFRSF18 | 0.007612 | 0.833053 | 0.728475 | 0.952644 | -0.26352 | Protective |
| IRF7 | 0.030816 | 0.837818 | 0.713507 | 0.983787 | -0.25529 | Protective |
| ARID5A | 0.005035 | 0.77482 | 0.648301 | 0.926031 | -0.36807 | Protective |
| TRIP6 | 0.035388 | 0.846652 | 0.72503 | 0.988677 | -0.24016 | Protective |
| MZT2A | 0.004305 | 0.761137 | 0.63108 | 0.917997 | -0.39377 | Protective |
| MAP1LC3A | 0.036294 | 0.827336 | 0.692817 | 0.987972 | -0.27346 | Protective |
| THEM6 | 0.002742 | 1.338273 | 1.105959 | 1.619386 | 0.420372 | Risk |
| MZT2B | 0.016418 | 0.798024 | 0.663712 | 0.959516 | -0.3255 | Protective |
| JUND | 0.030507 | 0.813352 | 0.674522 | 0.980757 | -0.29805 | Protective |
| CADM4 | 0.017485 | 0.812764 | 0.685022 | 0.964326 | -0.29909 | Protective |
| ANO9 | 0.02655 | 0.781611 | 0.628686 | 0.971735 | -0.35548 | Protective |
| TMEM160 | 0.049136 | 0.806716 | 0.651319 | 0.999187 | -0.30987 | Protective |
| C6orf226 | 0.023333 | 0.775068 | 0.62188 | 0.965991 | -0.3676 | Protective |
| PPP1R35 | 0.045085 | 0.802087 | 0.646459 | 0.995181 | -0.31817 | Protective |
| DDAH2 | 0.021336 | 0.793543 | 0.651722 | 0.966225 | -0.33362 | Protective |
| RPS15 | 0.027238 | 0.794098 | 0.647143 | 0.974425 | -0.33261 | Protective |
| ZNF219 | 0.011578 | 0.747068 | 0.59573 | 0.936851 | -0.42069 | Protective |
| COPS9 | 0.024642 | 0.780964 | 0.629471 | 0.968915 | -0.35667 | Protective |
| HGH1 | 0.039495 | 1.252334 | 1.01088 | 1.551461 | 0.32462 | Risk |
| ANAPC11 | 0.047845 | 0.79015 | 0.625735 | 0.997767 | -0.3398 | Protective |
| NUDT14 | 0.003901 | 0.714935 | 0.569241 | 0.897918 | -0.48412 | Protective |
| CEP131 | 0.031698 | 0.782556 | 0.625693 | 0.978745 | -0.35373 | Protective |
| SNAPC2 | 0.00954 | 0.733244 | 0.579906 | 0.927127 | -0.44764 | Protective |
| GPAA1 | 0.038039 | 1.28146 | 1.013778 | 1.619822 | 0.357789 | Risk |
| MICALL2 | 0.049603 | 0.780664 | 0.609698 | 0.99957 | -0.35723 | Protective |
| PPFIA3 | 0.016898 | 1.290988 | 1.046929 | 1.591942 | 0.368475 | Risk |
| DUS1L | 0.028681 | 0.762562 | 0.598154 | 0.972161 | -0.39107 | Protective |
| SNRNP70 | 0.022049 | 0.750807 | 0.587454 | 0.959584 | -0.41349 | Protective |
| HEXD | 0.040403 | 0.763301 | 0.589552 | 0.988256 | -0.38968 | Protective |
| ARHGAP39 | 0.039552 | 1.285092 | 1.012067 | 1.63177 | 0.361871 | Risk |
| PARD6A | 0.021892 | 0.755555 | 0.594535 | 0.960184 | -0.40439 | Protective |
| RPS9 | 0.003457 | 0.72106 | 0.579114 | 0.897798 | -0.47181 | Protective |
| RFNG | 0.02987 | 0.743812 | 0.56946 | 0.971544 | -0.42699 | Protective |
| DGAT1 | 0.031585 | 1.3282 | 1.025357 | 1.720489 | 0.409473 | Risk |
| FBXO6 | 0.022374 | 0.761877 | 0.603289 | 0.962155 | -0.39237 | Protective |
| KCTD17 | 0.031542 | 0.743294 | 0.567176 | 0.974099 | -0.428 | Protective |
| UBXN1 | 0.005269 | 0.726111 | 0.579913 | 0.909165 | -0.46174 | Protective |
| SART1 | 0.035775 | 0.786995 | 0.629299 | 0.984209 | -0.34557 | Protective |
| RAB13 | 0.043967 | 0.776481 | 0.607059 | 0.993186 | -0.36498 | Protective |
| MIIP | 0.048308 | 0.782309 | 0.613126 | 0.998176 | -0.35419 | Protective |
| TRMT2A | 0.022353 | 0.725667 | 0.551123 | 0.95549 | -0.46262 | Protective |
| CLASRP | 0.024414 | 0.741164 | 0.570986 | 0.962063 | -0.43213 | Protective |
| AGTRAP | 0.032431 | 0.765769 | 0.599652 | 0.977904 | -0.38502 | Protective |
| SERTAD1 | 0.036319 | 0.764753 | 0.594928 | 0.983057 | -0.38693 | Protective |
| LAMTOR4 | 0.006645 | 0.714946 | 0.561098 | 0.910979 | -0.48409 | Protective |
| PMF1 | 0.04213 | 0.742871 | 0.557722 | 0.989485 | -0.42882 | Protective |
| ARHGEF1 | 0.003626 | 0.67472 | 0.517599 | 0.879536 | -0.56764 | Protective |
| ZDHHC8 | 0.009222 | 0.70385 | 0.540343 | 0.916833 | -0.50666 | Protective |
| CCDC12 | 0.033928 | 0.747971 | 0.571928 | 0.978203 | -0.41894 | Protective |
| DNAJC4 | 0.048803 | 0.763619 | 0.58394 | 0.998586 | -0.38908 | Protective |
| ERF | 0.007446 | 0.698318 | 0.536839 | 0.90837 | -0.51804 | Protective |
| ENDOV | 0.007879 | 0.652317 | 0.475995 | 0.893954 | -0.61635 | Protective |
| MTA1 | 0.02203 | 0.704803 | 0.522425 | 0.95085 | -0.50471 | Protective |
| PICK1 | 0.033474 | 0.72961 | 0.545626 | 0.975633 | -0.4548 | Protective |
| DLAT | 0.02045 | 1.392457 | 1.052465 | 1.842281 | 0.477632 | Risk |
| PCYT2 | 0.010465 | 0.68578 | 0.513771 | 0.915376 | -0.54418 | Protective |
| CCDC9 | 0.037294 | 0.764195 | 0.593311 | 0.984297 | -0.38799 | Protective |
| PNKP | 0.037324 | 0.73924 | 0.556255 | 0.98242 | -0.43589 | Protective |
| SLC19A1 | 0.023706 | 1.389606 | 1.044895 | 1.848039 | 0.474676 | Risk |
| EIF3G | 0.037002 | 0.743471 | 0.562719 | 0.982283 | -0.42765 | Protective |
| SIRT7 | 0.02169 | 0.683757 | 0.494256 | 0.945913 | -0.54845 | Protective |
| CCDC28B | 0.005933 | 0.658601 | 0.489125 | 0.886797 | -0.60252 | Protective |
| RING1 | 0.013212 | 0.676916 | 0.497161 | 0.921663 | -0.56295 | Protective |
| PRKRIP1 | 0.027076 | 0.700185 | 0.510463 | 0.960421 | -0.51419 | Protective |
| USF2 | 0.014064 | 0.691176 | 0.514702 | 0.928156 | -0.53288 | Protective |
| MEA1 | 0.011796 | 0.676403 | 0.498942 | 0.916982 | -0.56405 | Protective |
| PANX1 | 0.016476 | 1.461377 | 1.071784 | 1.992587 | 0.547328 | Risk |
| DMAP1 | 0.015691 | 0.656183 | 0.466219 | 0.923549 | -0.60783 | Protective |
| EML3 | 0.019747 | 0.690769 | 0.506111 | 0.942799 | -0.53373 | Protective |
| MBLAC1 | 0.014425 | 0.653115 | 0.464272 | 0.918769 | -0.61459 | Protective |
| CHMP6 | 0.022102 | 0.711623 | 0.531762 | 0.952319 | -0.49081 | Protective |
| SEC63 | 0.000767 | 1.52993 | 1.194236 | 1.959985 | 0.613465 | Risk |
| BTBD6 | 0.00119 | 0.585068 | 0.423096 | 0.809047 | -0.77332 | Protective |
| ZBTB48 | 0.032088 | 0.684269 | 0.48367 | 0.968066 | -0.54736 | Protective |
| AKAP8L | 0.03039 | 0.690977 | 0.494457 | 0.965602 | -0.53329 | Protective |
| IFT43 | 0.037849 | 0.717172 | 0.524021 | 0.981519 | -0.47961 | Protective |
| ZNF672 | 0.033901 | 0.692498 | 0.49314 | 0.972449 | -0.53012 | Protective |
| MARCHF2 | 0.025468 | 0.702666 | 0.515597 | 0.957607 | -0.50909 | Protective |
| DAXX | 0.003304 | 0.604503 | 0.432079 | 0.845736 | -0.72618 | Protective |
| SGSH | 0.028015 | 0.671437 | 0.47063 | 0.957923 | -0.57468 | Protective |
| TYK2 | 0.034368 | 0.7004 | 0.503591 | 0.974125 | -0.51375 | Protective |
| XRCC1 | 0.002616 | 0.630489 | 0.466894 | 0.851405 | -0.66546 | Protective |
| VTA1 | 0.001831 | 1.648489 | 1.203793 | 2.257462 | 0.721144 | Risk |
| ZBTB17 | 0.024782 | 0.692255 | 0.50211 | 0.954405 | -0.53063 | Protective |
| CPSF4 | 0.009393 | 0.603698 | 0.412505 | 0.883507 | -0.7281 | Protective |
| THAP8 | 0.015201 | 0.64233 | 0.449309 | 0.918272 | -0.63861 | Protective |
| SNRNP35 | 0.02306 | 0.680846 | 0.488714 | 0.948513 | -0.5546 | Protective |
| PRKD2 | 0.039913 | 0.700631 | 0.498998 | 0.983739 | -0.51327 | Protective |
| PEX10 | 0.008382 | 0.604032 | 0.41523 | 0.878681 | -0.7273 | Protective |
| COPS6 | 0.040825 | 0.701493 | 0.499426 | 0.985316 | -0.5115 | Protective |
| ERCC1 | 0.010016 | 0.621813 | 0.433131 | 0.892689 | -0.68545 | Protective |
| LZTR1 | 0.023472 | 0.639721 | 0.434668 | 0.941507 | -0.64449 | Protective |
| MAGT1 | 0.004772 | 1.575597 | 1.148987 | 2.160604 | 0.655899 | Risk |
